# MEF-2 isoforms' (A-D) roles in development and tumorigenesis

**DOI:** 10.18632/oncotarget.26763

**Published:** 2019-04-12

**Authors:** Kiran Madugula, Ria Mulherkar, Zafar K. Khan, DeGaulle I. Chigbu, Dip Patel, Edward W. Harhaj, Pooja Jain

**Affiliations:** ^1^ Department of Microbiology and Immunology, the Institute for Molecular Medicine and Infectious Disease, Drexel University College of Medicine, Philadelphia, PA 19129, USA; ^2^ Department of Microbiology and Immunology, Penn State College of Medicine, Hershey, PA 17033, USA

**Keywords:** MEF-2, HDACs, HDACi, HTLV-1, ATLL

## Abstract

Myocyte enhancer factor (MEF)-2 plays a critical role in proliferation, differentiation, and development of various cell types in a tissue specific manner. Four isoforms of MEF-2 (A-D) differentially participate in controlling the cell fate during the developmental phases of cardiac, muscle, vascular, immune and skeletal systems. Through their associations with various cellular factors MEF-2 isoforms can trigger alterations in complex protein networks and modulate various stages of cellular differentiation, proliferation, survival and apoptosis. The role of the MEF-2 family of transcription factors in the development has been investigated in various cell types, and the evolving alterations in this family of transcription factors have resulted in a diverse and wide spectrum of disease phenotypes, ranging from cancer to infection. This review provides a comprehensive account on MEF-2 isoforms (A-D) from their respective localization, signaling, role in development and tumorigenesis as well as their association with histone deacetylases (HDACs), which can be exploited for therapeutic intervention.

## INTRODUCTION

Myocyte enhancer factor (MEF)-2 is a member of the MCM+Agamous+Deficiens+Serum response factor (MADS) box group of transcription factors. MEF-2 transcription factors have been well studied in the context of various developmental programs, including those of skeletal muscle, cardiac muscle, and neural tissue [1]. There are 4 isoforms of MEF-2, namely MEF-2A, MEF-2B, MEF-2C, and MEF-2D. These isoforms play an important role in embryogenesis and other epigenetic modifications that control gene expression [2–14]. Initially studied in the context of muscle development; involvement of MEF-2 is now well established in B-cell development, T-cell differentiation and thymocyte selection. MEF-2 regulates calcium-dependent transcription of the Interleukin-2 (IL-2) gene in T lymphocytes, and down-modulating MEF-2 by siRNA in primary human T cells leads to the inhibition endogenous IL-2 transcription. Due to intrinsic involvement of MEF-2 in T-cell development and IL-2 transcription, this transcription factor has been associated with T-cell leukemias/lymphomas (TCLs) in addition to a variety of other cancers as will be discussed later for each isoform. There are four major subtypes of TCLs: human T-Cell Acute Lymphoblastic Leukemia/Lymphoma (T-ALL), Adult T-Cell Leukemia/Lymphoma and Peripheral T-cell Lymphoma/unspecified (PTCL/U) [15]. MEF-2 isoforms are transcriptionally dysregulated in T-ALL and possess non-rearranged T-cell receptors [15, 16]. Moreover, MEF-2 has been implicated in HTLV-1-induced ATLL [17], and is under clinical investigation in various trials of CTCL and PTCL by targeting via HDACi [18].

Over the years, research progress has altered our perception of MEF-2 as less of a conventional transcription factor involved in development, and more as a vital player in tumor and leukemogenesis. MEF-2 is not only implicated in development, regulation and carcinogenesis, but also in chronic viral infections such as Epstein-Barr virus (EBV) [19–22], Human immunodeficiency virus-1 (HIV-1) [23] and Human T-cell leukemia virus-1 (HTLV-1) [17]. In addition, MEF-2 has been implicated in muscle related parasitic infections such as Trichinella [24]. It can also act as an immune-metabolic switch that can regulate infection susceptibility in drosophila models [25]. Our investigations into MEF-2 and HTLV-1 [26–40] are now providing rationale towards potential therapeutic strategies against virally induced ATLL based upon MEF-2: HDAC interactions. Our lab was the first to describe MEF-2A as a regulator of viral gene expression in the pathogenesis of the retrovirus HTLV-1. HTLV-1 encodes an oncogenic protein in the pX region of the viral genome known as Tax. Tax directly binds to MEF-2A and together with various co-activating transcription complexes hijacks the host transcriptional machinery to increase viral gene expression [17, 28]. With the exception of this study, the roles of MEF-2 and its isoforms in the course of HTLV-1 infection remain largely unexplored. MEF-2 isoforms were reported as the direct gene target of EBV nuclear antigen 1 (EBNA1), which supports the survival of EBV infected B-lymphocytes. EBNA-1 bound to B-cell specific transcription factors can inhibit MEF-2B and also suppresses cell growth as well as the viability of latently infected EBV cells [21, 22]. Additionally, other groups reported that MEF-2B enhances the activity of the promoter Zp of viral trans-activator protein BZLF1 by approximately 17.8-fold and promotes viral reactivation from latency [21].

MEF-2 contains a highly conserved MADS domain in the N-terminus, approximately 55 amino acids. The adjacent MEF-2 domain (29 amino acids) is also evolutionarily conserved, and serves as the DNA binding domain, interaction region for other proteins, and a site for dimerization. The MEF-2 domain acts as a docking site for various coactivators such as p300, CREB, etc., and corepressors including CABIN1, class IIa HDACs (histone deacetylases) and some kinases like GSK3β and other selective regulators like Protein Kinase A (PKA) [6, 8, 41, 42]. The transcriptional program of MEF-2 involves interactions with various coactivators such as p300, CBP, cFOS, cJUN, and ATF [43–52]. Another negative regulator of MEF-2 is Glycogen synthase kinase 3β (GSK3β) which has an indirect role of regulation of suppression of MEF-2 by modulation of p38MAPK. Pharmacological inhibition of GSK3β resulted in the increased activity of MEF-2(A-D) expression in myoblasts and cardiac myocytes. In-silico analysis showed consensus sites for GSK3β on MEF-2 as (S/T) XXX(S/T), although *in vitro* kinase assay stated that MEF-2A is only a weak substrate. Heart specific knockout of GSK3β in mice resulted in the upregulation of p38MAPK activity, suggesting the GSK3β as a negative regulator of MEF-2 isoforms and suggested crosstalk between the P38MAPK and GSK3β [53]. In cultured cerebellar neurons, a non-competitive inhibitor of GSK3β, inhibited caspase-3 activation and chromatin condensation but removing the depolarizing potassium and serum. Also, Lithium decreased MEF-2D hyperphosphorylation and apoptosis induced by calcineurin inhibition under depolarizing conditions. This suggests that GSK-3β phosphorylates and inhibits pro-survival activity of MEF-2D in cerebellar granular neurons [54]. GSK3β has been implicated in neuronal death and increase in its activity can induce the neurodegeneration and Alzheimer's disease. It has been stated that phosphorylation of MEF-2D at three specific residues in the transactivation domain inhibits MEF-2D transcriptional activity. Overexpressing a MEF-2 mutant resistant to GSK3β inhibition protected cerebellar granular neurons survival, stating the more suppressive role of GSK3β role in MEF-2 transcriptional activity [55]. In cardiomyocytes, CaMKII promotes hypertrophy and pathological remodeling by phosphorylating HDAC4 and subsequent activation of MEF-2. Protein kinase A (PKA) overcomes CaMKII mediated activation and selectively activates MEF-2 by regulated proteolysis of HDAC4. PKA degrades the N-terminal of HDAC4(HDAC-NT), which selectively inhibits the MEF-2 domain but not the SRF, thereby antagonizing the prohypertropic activity of CaMKII, without causing any effect on the cardiomyocyte survival and aiding in the cardio-protection and other cellular processes [56]. Although in certain studies it has been claimed that activation of PKA elevates the intracellular levels of cyclic AMP (cAMP) and inhibits skeletal myogenesis and this suggests MEF-2D as primary target of PKA and represses the transactivation of MEF-2D, but enhanced accumulation of HDAC4-MEF-2 complex inhibits the skeletal muscle differentiation [57]. It has been also shown that in embryonic day 18 (E18), Sprague Dawley hippocampal neurons, with the experimental induction of cAMP/PKA signaling promoted apoptosis. Also, Krüppel- like factor 6 (KLF6) was a transcriptional target of MEF-2 hippocampal neurons and knockdown of KLF6 antagonized the pro-survival role of MEF-2D and caused neuronal cell death [58]. HDACs are important and well characterized transcriptional partners of MEF-2, which have been exploited for therapeutic intervention using HDAC inhibitors (HDACi) to modulate the transcriptional machinery via the HDAC: MEF-2 axis. There are 18 categories of HDACs classified on the basis of their homology with yeast transcriptional regulator RPD3 and other biochemical properties [59]. The histone tails and their interactions with the DNA that control their modifications lead to activation or repression of gene transcription. Of these HDACs, classes I, II, and IV are zinc dependent and class III is NAD+ dependent. Class I HDACs [1–3, 8] are expressed ubiquitously in human cell lines and tissues, and are predominantly expressed in the nucleus. The class II HDACs can be defined into two subgroups IIA and IIB, which comprise HDAC4, 5, 7, and 9, and HDAC6, and 10 respectively, and they tend to shuttle between the nucleus and the cytoplasm. The third major group of HDACs consist of Class III HDACs and are also known as Sirtuins (SIRT1-7); at present their subcellular localization and tissue-specific properties if any, are not fully known. Class IV HDACs consists of only the most recently discovered HDAC11, and shows homology with both classes I and II [22, 60–70]. Class IIa HDACs are involved in the direct binding and suppression of MEF-2 proteins through the MADS/MEF-2 domains in the N-terminus. Association of class II HDACs with MEF-2 results in the deacetylation of histones in the vicinity of MEF-2 DNA-binding sites and subsequent suppression of MEF-2 target genes.

Many HDACs are a part of multiprotein complexes and interact with various proteins that affect their activity and specificity, and are controlled by various post-translational modifications such as phosphorylation, acetylation, and sumoylation [71, 72]. HDACs are constitutively bound with co-repressor complexes including HDAC1: SIN3, NURD and HDAC3: NCOR1 (nuclear receptor co-repressor 1) and SMRT (silencing mediator of retinoic acid and thyroid hormone receptor) [73]. The class IIa HDACs form multiprotein complexes with MEF-2, NCOR1 and SMRT along with association with HDAC3 for regulation of its deacetylase activity. The nuclear co-repression of the various transcription factors is regulated by phosphorylation events which promotes the binding of 14-3-3 via G-protein coupled receptors, and results in their shuttling back to the cytosol thus allowing for reactivation of the target genes [74]. HDACs not only target histone moieties but target non-histone targets as well, thereby enhancing the diverse regulatory properties of PTMs. There are lysine acetylation signatures that have been identified not only on nuclear proteins but also on cytosolic and mitochondrial proteins across many different species [75]. More recently, the MEF-2: HDAC axis has also been implicated in various cancers. Increased expression of class IIa HDACs has been correlated with suppression of MEF-2 transcriptional activity and poor prognosis of estrogen receptor-positive (ER+) breast tumors as well as increased proliferation of mammary epithelial cells [76]. Overexpression of HDAC9 has been linked to poor prognosis of oral squamous cell carcinoma by targeting MEF-2D and repressing expression of MEF2-dependent genes [77]. Class IIa HDACs in conjunction with MEF-2 have been implicated in leiomyosarcomas as well [78].

Because of their widespread influence on oncogenesis, HDACs have emerged as the target of an entire class of anticancer drugs collectively known as HDACi (Histone deacetyalase inhibitors) that encompass a diverse group of small molecule drugs that can induce apoptosis, cell cycle arrest, differentiation, and autophagy of cancer cells and promote anti-angiogenic effects [79–86]. These drugs are mostly efficacious for treatment of leukemias, and have not shown great success against solid tumors [83, 86]. MEF-2 proteins also play a key role in T-cell survival by interacting with calcium-regulated NFAT family members, p300, and p/CAF [87–91]. This review will provide a detailed discussion on the signaling pathways that control the activation of different isoforms of *MEF-2* via various signaling molecules and cascades, and their implications in various disease phenotypes. Furthermore, this review will also provide a glimpse of the potential of HDACi in the context of MEF-2-based therapeutics.

## REGULATION OF MEF-2

**Figure 1:**
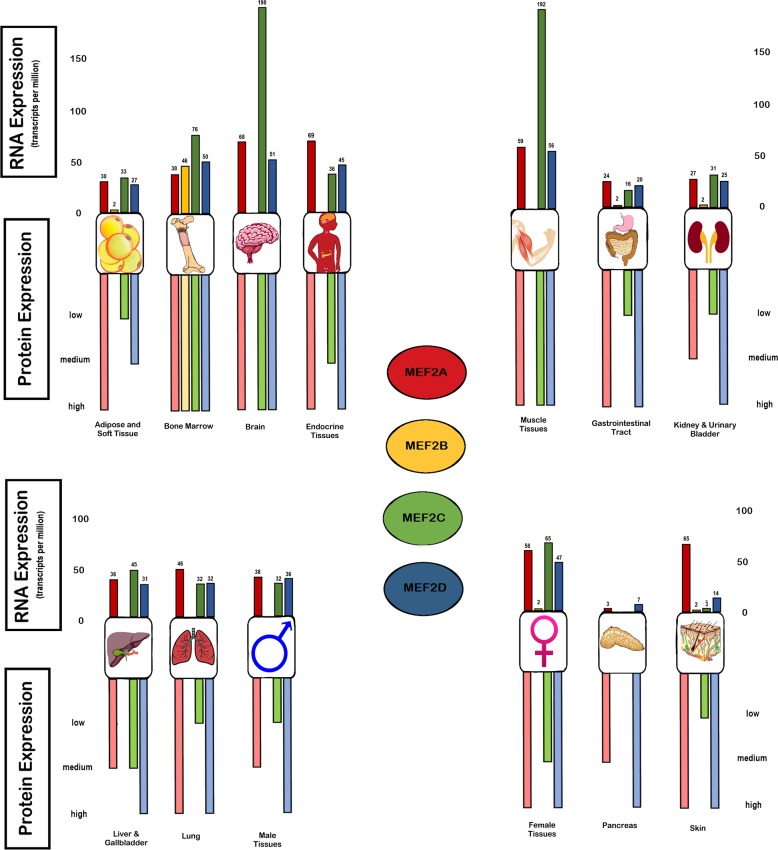
Basal expression of MEF-2 isoforms in organ systems and tissues in RNA and protein The expression of MEF-2 isoforms are illustrated in the various organ systems at the transcriptome level in TPM (transcripts for kilobase million) via RNA-seq expression and the protein expression from high to low levels which is obtained from the immune-cytochemistry quantification (https://www.proteinatlas.org/).

**Figure 2:**
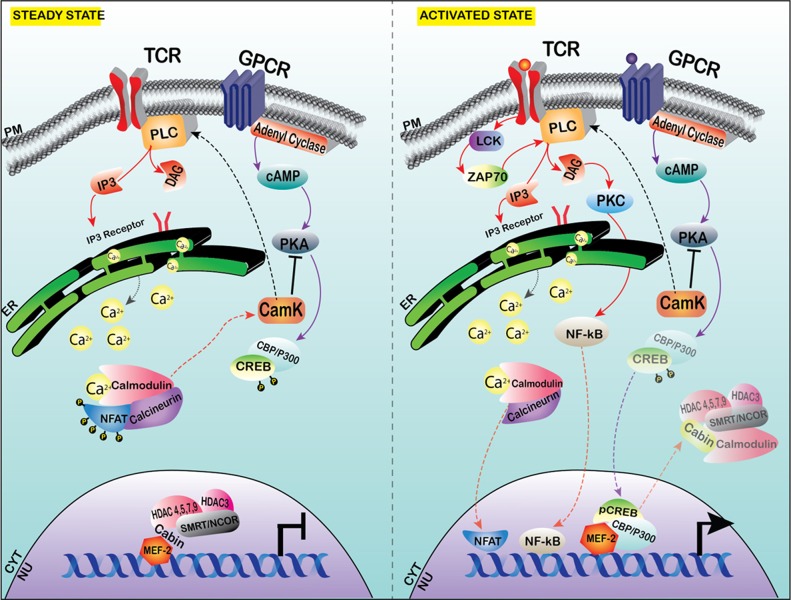
Calcium signaling affecting MEF-2 transcription at steady state versus the activated state in T cells MEF-2 is constitutively bound with co-repressor complex, which comprises of CABIN-1, HDAC3 and Class II HDACs along with SMRT/NCOR in steady state. With the activation of stimulus, calcium stores from the ER are released into the cytoplasm via the IP3 receptor activation. The Ca2+ activates the calmodulin-calcineurin complex, which dephosphorylates the inactive NFAT in the cytoplasm into activated NFAT and translocates into the nucleus (this has been observed in depolarized neurons and skeletal myoblasts in specific isoforms of MEF-2). In T-lymphocytes, Calcium influx activates the CamK and PKA, which allows the transcription of NF-ĸB. PKA phosphorylates CREB and pCREB/P300 complex translocates to the nucleus and the co-repressor shuttles out of the nucleus and allows the activation of MEF-2, in the proliferation of T cells (shown in Jurkat T-cell line *in-vitro*).

**Figure 3:**
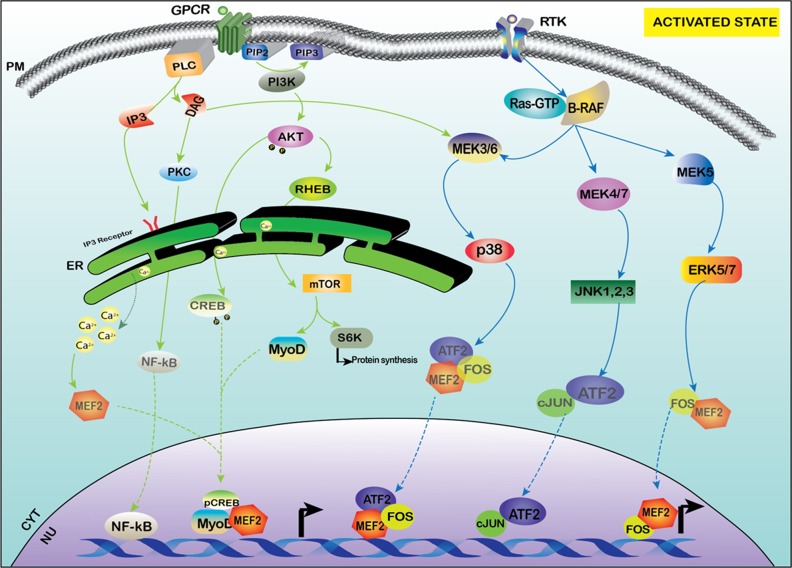
Activation of MEF-2 by PI3K-AKT and MAPK signaling MEF-2 is also activated by PI3K/AKT pathway which activates mTOR as a downstream target and also increases the transcription of MyoD and MEF-2 along with other transcription factors. The receptor tyrosine kinase (RTK) under specific stimulus activates various different transcription factors of AP-1 family like c-JUN and FOS and aids in the activation of MEF-2 via the MAPK family of MEK/ERK.

MEF-2 family proteins act as effector molecules for multiple signaling cascades, with the major regulators of their signaling being activated and guided by multiple pathways through various second messenger and signaling molecules. Various activators of MEF-2 activity include Calcium (Ca2+)-mediated signaling, Mitogen activated protein signaling (MAPK), Wnt signaling and the PI3K/AKT signaling pathways. The basal expression in various tissues and cells will be illustrated in Figure 1 and a detailed outline of the key regulatory molecules as well as the signaling pathways are provided in Figures 2 and 3 of this review.

### Calcium mediated regulation

Calcium is a critical second messenger in various cellular processes in the cell. The major calcium/calcineurin responsive elements present in the NUR77/NR4A1 promoter act as docking sites for MEF-2 transcription factors. NFAT (Nuclear Factor in activated T cells) is activated by calcium influx, and in turn interacts with *MEF-2D* for its activation via calcineurin. NF-κB becomes activated when stimulation of the T-cell receptor results in calcium and protein kinase-mediated signaling that activates the IκB kinase (IKK) complex which then phosphorylates inhibitory κB proteins (IκBs), triggering their degradation by the proteasome and thus allowing NF-κB to translocate to the nucleus. The calcineurin-mediated dephosphorylation of MEF-2 at residue S408 activates the switch from sumoylation to acetylation at lysine-408, leading to the inhibition of the differentiation of the dendritic claw [92]. Furthermore, Calcineurin binding protein 1 (CABIN1), MEF-2-interacting transcriptional repressor (MITR), and HDAC4 are repressors of MEF-2 activity. CABIN-1, together with MITR and HDAC4, have been shown to repress MEF-2 transcriptional activation by binding to MEF-2 constitutively. HDAC4 and MITR contain calmodulin binding domains that are similar to the MEF-2 binding domain; in response to an influx of calcium, calmodulin binds to Cabin-1 and HDAC4 in turn releases MEF-2 and derepresses its transcriptional activity [47]. T cells expressing a truncated mutant of CABIN1, devoid of the C-terminus, serves as the binding domain for MEF-2 and calcineurin, showed significant increase in the release of cytokines such as IL-2, IL-13, IL-4 and IFN-γ [93]. Calreticulin represents another chaperone protein in the calcium signaling to MEF-2. Calreticulin is located in the ER/SR (endoplasmic/sarcoplasmic reticulum), and modulates the intracellular Ca2+ stores and associated signaling events. Calreticulin is known to control myocardial development; in the absence of Calreticulin (*Crt*^−/−^), there is impaired nucleocytoplasmic transport of the cardiac transcription factor MEF-2C [94], and myofibrillogenesis is impeded in a calcium dependent manner. The Ca^2+^/calmodulin-dependent protein kinase IIδ2 (CaMKIIδ2) is a positive regulator of vascular smooth muscle cells (VSMCs) dedifferentiation from contractile to synthetic phenotype in chronic vascular diseases such as atherosclerosis, that contribute to the growth of neointima. MEF-2 is a transcriptional activator of the VSM phenotype and regulated in an HDAC-dependent manner, and suppression of CaMKIIδ2 decreased the DNA binding affinity of MEF-2 and down regulation of its target genes Nur77 and MCP1n [90]. Calpain-3 (Capn-3) is a calcium-dependent proteolytic enzyme which also regulates certain non-proteolytic functions. *MEF-2A* has been implicated in regulating *Capn3* gene expression by occupying the promoter region of the Capn3 gene in rat denervated gastrocnemius muscle tissue, positively correlated with the increase of *MEF-2A* expression in protein and RNA expression in L6 myoblasts [95]. Calcium is known to exert an important role in the survival and proliferation of ATLL cells and NFAT family members have been found to be activated in HTLV-1 transformed cells [96]. In HTLV-1-infected cells, the viral transactivator protein Tax interacts with a number of transcription factors and proteins to stimulate T-cell proliferation, many of them regulated by calcium [97].

In HTLV-1-infected cells, Tax-mediated activation of histone acetylase p300, CRE-binding protein (CBP), and p300/CBP associated factor (p/CAF), among others, results in transcriptional activation and T-cell proliferation [17]. For instance, Tax-mediated activation of NF-κB results in expression of interleukin-2 (IL-2)/IL-2 receptor (IL-2R), IL-9, IL-13, IL-15/IL-15R, and receptors of the tumor necrosis factor family, thereby promoting proliferation as well as transformation of T cells [97]. MEF-2 proteins also play a key role in regulating T-cell survival by associating with proteins including p300, p/CAF, and NFAT and are regulated by calcium [17]. Cabin1, MITR, and HDAC4 are repressors of MEF-2 activity that are displaced by the calcium/calmodulin complex [98]. Hypercalcemia is observed in 31% of ATL patients at disease onset [99]. About 70% of ATL patients develop hypercalcemia, particularly in the aggressive stage of the disease [100]. Calcium levels are often elevated in acute and lymphoma forms of ATL, but not in chronic and smoldering forms [101]. Hypercalcemia is present in about 50% of acute type ATLL patients at diagnosis, and it may develop in another third of patients as the disease progresses [101]. In contrast to other leukemia cells, ATL cells show enhanced growth by addition of calcium in a dose-dependent manner and inhibited growth in response to calcium antagonists and calmodulin inhibitors [102].

### MAPK (P38/ERK/JNK) signaling

The other signaling pathways associated with *MEF-2* proteins are mitogen-activated protein kinase (MAPK) signaling pathways which control various cellular processes such as proliferation, differentiation [103, 104], cell cycle, survival and apoptosis as reviewed in [105]. MAPKs are serine/threonine kinases that can target both cytoplasmic and nuclear substrates. In mammals, there are four canonical MAPK pathways: P38 mediated signaling, extracellular signal regulated kinase signaling (ERK1/2/5), non-mitogen mediated signaling and c-Jun kinase/stress-activated protein kinase (JNK/SAPK) signaling [103, 104, 106]. The important phosphorylation nuclear targets of p38 include ATF1/2/6, p53, MEF-2, and ELK-1 [103, 104]. p38-MEF-2 signaling is involved in the proliferation and differentiation of B cells [107, 108], and also mediates the survival of cerebellar granular neurons [109, 110]. It has been further implicated in various types of developmental pathways in the context of regulation with the MEF-2 family and regulates various signaling cascades in cardiomyocyte biology [111]. Studies have shown close correlation between p38 MAPKs, calcium-calmodulin dependent protein kinases and calcineurin signaling pathways that activate the expression of myogenesis via activating MEF-2 transcription factors [112]. The presence of high glucose significantly increased p38 MAPK signaling and escalated cardiac hypertrophy induced by hyperglycemia, whereas miR-373 inhibits *MEF-2* activity by down regulating p38 signaling and reducing glucose induced hypertrophy [113]. The activation of p38 MAPK results in the degradation of HDAC4 followed by the release of RUNX2 and activation of MEF-2 genes in modulating chondrocyte hypertrophy [114]. The phosphorylation of p38 MAPK results in the interaction of β-catenin and *MEF-2* in primary vascular smooth muscle cells (VSMCs) via increased β-catenin nuclear retention and increased activation of *MEF-2* mediated Wnt/β-catenin signaling for cell proliferation [115]. The activation of *MEF-2* via a p38-dependent pathway causes vascular inflammation through the activation of MCP-1 in VSMCs and allows the infiltration of macrophages into the endothelium [116]. *MEF-2* also plays an important role in the Alzheimers disease, specifically amyloid precursor protein (APP)-mediated signaling pathway by inhibiting neuronal apoptosis by mediating the p38 MAPK-dependent pathway and activation of MEF-2-dependent gene transcription; alterations in this pathway results in the neuronal degeneration that occurs in Alzheimer's disease (AD) [117].

There is a unique MEF-2 interacting domain in the C-terminus of ERK5, and mice lacking ERK5 or MEK5 exhibit cardiovascular defects during embryogenesis [118]. ERK5 also activates members of the MEF-2 family during the process of monocytic differentiation of myeloid leukemia cells [119]. Mouse embryonic fibroblasts that lack ERK 1/2 completely fail to proliferate [120]. The sheer stress-induced factor Krüppel like factor- 2 (KLF-2) is transcriptionally induced by a MEK5/ERK5/MEF-2-MAPK pathway which indirectly activates RhoA which causes the formation of actin shear fibers that control the alignment of flow and also inhibits the JNK/c-Jun/ATF signaling pathway in the vascular endothelial homeostasis [121]. It has also been shown that MEF-2 is activated via the MEK5-ERK5 pathway by Hepatitis C non-enveloped core like particles (HCVne) for altering biological activity [122]. MEF-2 and miR-193b-3p and miR-203a have direct biological association in downstream p38 signaling and are implicated as dysregulated genes in colorectal carcinoma [123, 124]. DNA damage induced apoptosis suppressor (DDAIS) is a protein that promotes cancer cell survival via its anti-apoptotic activity and is activated by NFAT. In response to EGF (Epidermal growth factor) stimulation, the ERK5/MEF-2 pathway subsequently induces DDIAS expression to promote the invasion of cancer cells by activation of various β-catenin target genes [125].

### Post-translational modifications associated with MEF-2

The regulation of MEF-2 is also associated with the degradation pathways involved in the cell cycle. It has been reported that MEF-2C is degraded in the mitotic phase of proliferating cells by Anaphase Promoting complex/Cyclosome (APC/C), this downregulation is necessary for the efficient progression of the cell cycle checkpoints. This mechanism requires the presence of 2 phospho-motifs, pSer98 and pSer110, which mediate the interaction of CDC20 and MEF-2C for this process to occur and can mediate the cell proliferation [126]. In primary cerebellar granular neurons, Cyclin-dependent kinase5(CDK5) induces neurotoxic effects by phosphorylating and degrading MEF-2 via a caspase dependent manner. Neurotoxic conditions results in the nuclear activation of Cdk5 activity, which in-turn resulted in the activation of caspase3 and subsequently apoptosis [127]. Moreover, in other studies it has been reported that CDK5 also stimulated the sumoylation. It has been shown that Ser-444 of MEF-2D is required for the sumoylation of Lys-439. HDAC4 stimulated the modification by acting through Ser444. Opposing this inhibitory function over MEF-2D, Calcineurin also known as protein phosphatase 2B (PP2B) dephosphorylated Ser-444 and inhibited sumoylation of Lys-439 thereby activation of MEF-2 [128]. The transcriptional activity of MEF-2A is highly repressed by sumoylation, there are SENPs (Sumo specific proteases) which acts as an de-sumoylation enzyme of MEF-2A. The expression of SENP2 markedly increased the activity of MEF-2A in neuroblastoma cell lines and it mediated the activity dependent regulation of MEF-2A based on the stimuli, thus playing an important role in the activation dynamics of MEF-2A [129]. Neuronal survival and activity are controlled by MEF-2 isoforms and they exhibit a differential stress response to various stimuli and certain isoforms of MEF-2 are activated more than the others. It has been shown that MEF-2A but not MEF-2C or MEF-2D was modified in dopaminergic neuronal cell lines. It has been shown that MEF-2A is the only isoform that is ubiquitinated in the N’ terminus and also markedly reduced the DNA binding activity and transcriptional activation. Moreover, interfering the degradation of ubiquitinated MEF-2A induced neurotoxins associated with Parkinson's disease (PD in model animals suggesting the selective regulation of MEF-2 in ubiquitin-proteasome pathway [130]. Redox-mediated post translational modifications can act has a molecular switch in MEF-2 function. Nitric oxide (NO) mediates redox reaction called S-nitrosylation of MEF-2 which acts a redox switch to inhibit both neurogenesis and survival. Structural analysis showed the dimerization of MEF-2 creates a pocket and nitrosylation occurs on the evolutionarily conserved cysteine residue in the DNA binding domain, which disrupts the binding and transcriptional activity of MEF-2 and leads to impairment of neuronal survival and neurogenesis both *in-vitro* and *in-vivo* [131]. MEF-2 suppresses the excitatory synapse number by the degradation of the synaptic scaffold protein or post synaptic density protein 95 (PSD-95) degradation, a gene that is defective in the rodent model of Fragile X syndrome. MEF-2 induces a PP2A mediated dephosphorylation of murine double minute-2 (Mdm-2), which is an ubiquitin ligase for PSD-95 which translocates to the nucleus and degrades PSD-95 and causes synapse elimination [132]. MEF-2D is activated by calcium/calmodulin-dependent protein Calcineurin (CaN or PP2B). Both MEF-2D and CaN bind to the scaffold of muscle A-kinase anchoring protein β (mAKAPβ) which is localized in the nuclear envelope. This phenomenon decreases skeletal myoblast differentiation and inhibits neonatal rat ventricular myocyte hypertrophy. The formation of signalosomes by mAKAPβ is required for the dephosphorylation and desumoylation of MEF-2D in C2C12 cells. The decrease in MEF-2D phosphorylation switched the activation of p300 instead of type IIA histone deacetylases and caused ventricular myocyte hypertrophy, suggesting the role of post-translational modifications of MEF-2 in the formation of active and repressive transcriptional complexes with signalosomes [133].

### MEF-2 and its functions in mitochondria

MEF-2 and its isoforms play a critical role in mitochondrial maintenance and biogenesis and the phenomenon has been established in the various in-vitro and in-vivo models. A conserved region of MEF-2 gene, the MADS-box phosphorylation domain regulates the mitochondrial function in many cell types such as skeletal, cardiac and smooth muscle cells [134]. MEF-2 along with Serum response factor (SRF) regulate the expression of miR-133a and mitochondrial function via translational inhibition of mitophagy and cell modulating protein called NIX. In mice, MEF-2 also plays an important role in mitochondrial biogenesis. It has been shown that specific mi-RNAs, such as miRNA-27B regulates mitochondrial biogenesis in myocytes via targeting the fork-head box j3 (FoxJ3) and downregulated the expression levels of downstream targets like MEF-2c, PGC1α, NRF-1and mtTFA [135]. In xenopus models, mitochondria have been linked to the maintenance of synaptic and neuronal plasticity by integrated functions of MEF-2 and mitochondria. MEF-2 serves as a target of mitochondrial caspases and in-turn MEF-2 regulates the mitochondrial gene transcription essential for the production of hydrogen peroxide and superoxidase controlling the spatio-temporal neuronal plasticity [136]. It has also been shown that MEF-2D has a neuro-protective ability with neurotoxicity associated with the MPTP in Parkinson's disease [137]. This sort of neuroprotective activity is induced by agents like Methylene blue (MB) via MEF-2D and can ameliorate the neurochemical/neuropathological impairments in animal models of various neurodegenerative disorders. MB upregulated the expression of mitochondrial NADH dehydrogenase −6 (ND6) in a MEF-2D dependent manner, and knockdown of MEF-2D abolished the MB mediated increase of ND6 and the neuroprotective effect. Moreover, MB also induced the activation of AKT pathway and suppressed the GSK-3β activity which is an inhibitor of MEF-2D function [138]. Some anti-cancer agents like SU4312, yielded an unexpected neuroprotective effect by potentiating the pro-survival effect of PI3-K/AKT pathway to downregulate the inhibitory effect of MEF-2D inhibitor glycogen synthase kinase-3 beta (GSK3β) and ameliorated parkinsonian motor defects and restored levels of MEF-2D [137]. It has also been established that there is an association between PD and mitochondrial toxins, other environmental pesticides. In this study it has been shown that mitochondrial-toxin induced nitrosative/oxidative stress results in the S-nitrosylation of MEF-2C in A53T mutated cells compared to isogenic controls [139], via inhibiting the MEF-2C-PGC1α pathway and leading to mitochondrial dysfunction and apoptotic cell death. In certain murine models, it has also been shown that the activation of conventional Tcells and Tregs (T regulatory cells) led to the increase in gene expression of OXPHOS (Oxidative phosphorylation). The deletion of HDAC9, which is an inhibitor of MEF-2 increased the Treg suppressive activity as well as expression of Pgc1α and Sirt3 and improved mitochondrial respiration. The study shows that key regulators of OXPHOS are required for optimal functioning of Tregs and Treg dependent allograft acceptance [140].

### Mutations in MEF-2 in the context of diseases

Numerous studies have investigated the genetic aberrations of MEF-2 in various cancers [1, 141]. Mutations in the N-terminal domain lead to defects in DNA binding activity and impairs MEF-2 transcriptional activity [142]. The C-terminal domain, which acts as the regulatory or the catalytic domain, has 2 transactivation domains (TADs) which are highly divergent between the isoforms; they are also known to contain specific phosphorylation sites for multiple kinases, allowing for tight regulatory activity of the MEF-2 proteins [143]. The final few amino acids of the C-terminal region comprise the nuclear localization signal (NLS) for all the isoforms which is absent in the MEF-2B isoform, although MEF-2B can be found both in the nucleus and the cytoplasm [144]. The defects associated with the absence and/or aberrations of MEF-2 have been well characterized in murine models and humans. MEF-2 activity is coordinated by multiple layers of regulation and all the isoforms are subjected to alternative splicing events, post-translational modifications in the C-terminus, and dimerization capability with other transcription factors at the N-terminus. Mutations in these respective domains result not only in developmental abnormalities, but may also cause tumors, leukemias and aberrations in the transcriptional machinery. *MEF-2a* null mice exhibit pre/post-natal defects in cardiac development and muscle differentiation [145, 146]. However, mice deficient in MEF-2B do not have significant observable phenotypes, since the other isoforms can compensate for the loss of its function [147]. Several mouse models have been generated to study the abnormalities associated with loss of *MEF-2C*. In the absence of *MEF-2C* there were defects associated with B-cell proliferation and BCR stimulation, and a decrease in B-cell numbers in the bone marrow [148]. There was also a considerable loss of granulocyte progenitor function with a loss of function allele of *MEF-2c* [149]. Preliminary studies of *MEF-2d*-null mice indicated that these mice exhibited normal viability and life span, but heterozygous alleles of *MEF-2c* mouse showed debilitating effects in bone differentiation, and *MEF-2d* knock-out mice had blindness in photoreceptor cells within the retina [150].

### Isoforms of MEF-2

Table 1 summarizes some key information on MEF-2A, MEF-2B, MEF-2C, and MEF-2D. These isoforms play an important role in embryogenesis and other epigenetic modifications that control gene expression [13, 151–153]. The chromosomal coordinates of MEF-2 are 15, 19, 5 and 1 for the MEF-2A, 2B, 2C, and 2D, respectively [154]. The perception of MEF-2 as a developmental transcription factor has gradually shifted to encompass emerging findings of MEF-2 in more pathological processes such as tumorigenesis and involvement in viral pathogenesis/gene expression in HTLV-1 and activation of latent virus in the case of EBV [17, 19–22]. The basal RNA and protein expression and various aberrations of each isoform will be discussed in detail in the following sections and is illustrated in Figure 1.

**Table 1:** Expression, transcriptional activity and associated diseases of MEF-2 isoforms

Isoforms	Expression	Development	Mutations	Activity	Gene-targets	Disease
**MEF-2A**	Expressed in brain, endocrine tissue, lung, skin and soft tissue. Expressed in cardiac, skeletal and smooth muscle. Highly overexpressed in glioma, lymphoma, and melanoma.	Required for proliferation, differentiation and maturation of neural stem cells into neurons and glial cells [164, 165]. Muscle-regeneration and tissue repair [95]. Myofibroblast differentiation [158].	MEF-2A mutations is associated with premature myocardial infarction and coronary heart disease [172]. Also associated with reduced myofibroblast proliferation and differentiation [152].	Downregulation of Wnt signaling via miRNA/Gtl2-Dio3 loci [155]. Regulates miRNA-143 [156]. Regulates Calpain 3 gene expression [95]. Also, involved in immune response [180, 183, 184].	*MEF-2A* is located on chromosome 15q26 [154]. GSK3beta and Xirp2 are transcriptional target of MEF-2A [53–55, 161]. *Cited2* is a possible transcriptional target [155].	MEF-2A is involved in the pathogenesis of HTLV-1 and ATLL [18, 26–40]. Accelerates the progression of atherosclerosis [184]. Implicated in HCC and gastric cancer [180, 183].
**MEF-2B**	Expressed in tonsil, appendix, germinal center of lymph node, and bone marrow. Highly-expressed in lung carcinoma and lymphoma. Expressed in cardiac and skeletal muscle.	MEF-2B is required for the development of cardiac and skeletal muscle [189].	Mutation causes dysregulation of proto-oncogene BCL6 [193, 194]. Associated with leukemia and carcinoma [193, 195–197].	Promotes tumorigenesis [125].	*MEF-2B* is located on chromosome 19p12 [154]. ROS-induced NOX-1 signaling [188]. Binds in the promoter of DDAIS [125].	Involved in follicular lymphoma, Burkitt's lymphoma, mantle cell lymphoma, splenic marginal zone lymphoma, and diffuse large B cell lymphoma [196, 197]. Hypertension in VSMCs [188] and TGFβ-induced EMT and tumorigenesis [125, 191].
**MEF-2C**	Expressed in nucleoplasm and neuroplasmic vesicles. Expressed in cardiac, skeletal muscle and brain tissue (especially in cortical excitatory neurons). Expressed in both common myeloid & lymphoid progenitors. Maturing hematopoietic stem cells and B cells.	It is involved in the development of cardiac and skeletal muscle [152]. Development of lymphoid and myeloid progenitor cells [153]. Development of the cerebrocortex in its role in corticostriatal circuit [202].	Associated with decline in cognitive ability as well as in development of autism, schizophrenia, and epilepsy [10, 202–204]. Implicated in congenital heart defect and forelimb malformations [210, 221].	It possesses differential activity in membrane depolarization [201]. Required for neuronal differentiation and commitment of embryonic stem cells [199].	*MEF-2C* is located on chromosome 5q14 [154]. Target genes include GPM6A, DLx6, Hand2, Myogenin, and Myokinase [200, 207, 233].	MEF-2C implicated in DiGeorge syndrome [209].
**MEF-2D**	Highly expressed in cerebrocortex, endocrine system, skin, kidney, and breast. Present in hematopoietic stem cells and germinal centers of lymph node. Expressed in cardiac and skeletal muscles.	It is required for the development of neuronal and muscle cells [53, 54]. Terminal differentiation of neonatal cardiomyocytes and human macrophages [163, 52].	Mutation implicated in the inhibited differentiation of common lymphoid progenitors (differentiation into CD19 positive B-cells) [248].	Promotes the survival of dopaminergic neurons [242]. In GLUT-4 transcriptional activity [158, 182].	*MEF-2D* is located on chromosome 1q12-q23 [154]. *DYRK1A* is a target gene [240].	MEF-2D is involved in cancer of the colorectal tissue, breast, prostate, lung, liver, and thyroid [124, 125]. Implicated in melanoma [279–281], and pathobiology of B-cell acute lymphoblastic leukemia (B-ALL) [248]. Also, Parkinson's disease, cardiac myxoma, and lung cancer [239, 244, 250, 251].

### MEF-2A

MEF-2 genes are expressed in very distinct and specific temporo-spatial patterns. MEF-2A is transcriptionally autoregulated and is activated by Erk5 and p38 MAPK signaling; the *MEF-2a* protein product regulates transcription either positively or negatively [53]. MEF-2A is a DNA binding transcription factor which induces muscle development, neuronal differentiation, cell growth and apoptosis specific genes. It is located on chromosome 15q26 [130]. MEF-2A is present ubiquitously in all tissues but enriched in the brain, endocrine tissues, muscle, lungs, skin and soft tissues. In the CNS, at the transcriptome level, it is highly expressed at 68.3 TPM (transcript per kilobase million) cerebral cortex, but expressed at low levels in the hippocampal, caudate or cerebellar regions. However, elevated expression of MEF-2 protein is observed in the cerebral cortex and cerebellar region. In muscle tissues, such as cardiac, skeletal, and smooth muscle, MEF-2 has medium to high level expression at 59.2 TPM, 21.6 TPM, and 52.4 TPM, respectively. Higher levels of MEF-2A protein expression are seen in cardiac and skeletal muscles, but considerably less observed in the smooth muscle. In the bone marrow and immune system, it is also expressed considerably less (medium level) at both at the transcriptome and the protein level (12 TPM). MEF-2A was found to be highly overexpressed in gliomas, lymphomas and melanomas as well as lung cancers but not to a great extent (https://www.proteinatlas.org/).

### MEF-2A in muscle development

It has been suggested that MEF-2A is absolutely indispensable for proper myoblast differentiation compared to other isoforms. In bovine myoblasts, via regulation through myozenin2 (MyoZ2) [2], it has been shown to be a transcriptional regulator of *Calpain3* (*Capn3*) gene expression, which in turn controls L6 rat myoblast differentiation. Defects in this pathway might result in atrophy of the proximal limb muscles [95]. Skeletal muscle regeneration and repair are crucial for regeneration of diseased muscle. Injured *MEF-2a* knockout mice are impaired by myofiber defects and necrosis, a process controlled by MEF-2A via the microRNA (miRNA) Gtl2-Dio3 locus that downregulates Wnt signaling activity. This step is critical for proper muscle regeneration and tissue repair and may be dysregulated in muscular dystrophy. The same locus is involved in regulating *Cited2* in cardiomyocyte proliferation via MEF-2A through increased *VEGFA* activity [155]. Also, miR-143 is regulated by MEF-2A in hydrogen peroxide-induced senescence in vascular smooth muscle cells (VSMCs); overexpression of MEF-2A along with miR-143 shows synergy in senescence induction whereas the knockdown has opposite effects [156]. MEF-2A and AP-1 provide antagonistic regulation of Heat-shock protein B7 (HspB7) which has been implicated in muscular atrophy [157].

In diabetic mouse models, MEF-2A knockdown in cardiac fibroblasts led to significant reduction of hyperglycemia-induced cardiofibroblast proliferation, myofibroblast differentiation, and matrix metalloprotease (MMP) and collagen activity, as well as improved cardiac function and collagen deposition. This process was achieved via downregulation of Akt and TGF-β/Smad signaling, which can serve as a potential treatment modality in diabetic-induced cardiac remodeling. MEF-2A:MEF-2D heterodimers, responsible for GLUT-4 (Glucose transporter-4) transcriptional activity, were shown to be selectively decreased in insulin-deficient diabetic rats [158]. Protein Kinase B2 (AKT2) is involved in cardiomyocyte signaling, heart development and systemic blood pressure. AKT2 via EndoG, a mitochondrial-specific nuclease, regulates MEF-2A in the myocardium, and the deficiency of AKT2 results in defective myocyte development through the EndoG:MEF-2A signaling pathway [159]. Acute knockdown of MEF-2A results in defective integrity of costameres, a macromolecular complex responsible for transmission of contractile force through the straited muscle cells. This costamere defect results in abstruse malformations in myofibrils, defects in focal adhesion and adhesion-dependenT-cell death [160].

Xirp2 (also called CMYA3) is a cardiomyopathy-associated gene induced by Angiotensin-ii (Ang-ii), and a direct transcriptional target of MEF-2A. Both MEF-2A and Xirp2 serve as the important downstream regulators of Ang-II in mediating pathological cardiac remodeling [161]. MEF-2A is also implicated in cardiac hypertrophy and myopathy due to arsenic exposure in rats [162]. Another study suggests that glycogen synthase kinase3β (GSK3β), a suppressor of both myogenesis and cardiac hypertrophy, is a transcriptional target of MEF-2A. Inhibition of GSK3β results in the activation of the P38 MAPK pathway and subsequently MEF-2A activity that controls skeletal and muscle gene expression [53]. Also, inhibition of MEF-2A or MEF-2D results in activation of cell cycle genes, but the downregulation of terminal differentiation of neonatal rat cardiomyocytes [163].

### MEF-2A in neuronal development

All the MEF-2 isoforms are extensively but differentially expressed during phases of pre- and post-natal development. They follow distinct roles at various stages of the maturation of neurons, in neural stem cells that have an ability to proliferate and differentiate into various neuronal and glial phenotypes. *In vivo* analysis has implicated various MEF-2 transcription factors in synapse regulation and neuronal survival [164]. Studies indicate that MEF-2A is present in undifferentiated neural stem cells, and after subsequent differentiation exhibit higher amounts in neuronal cells. MEF-2A plays a critical role in differentiation and maturation of neural stem cells into neurons in rat models [165]. MEF-2 transcriptional activity is also activated by neurotrophins such as brain-derived neurotrophic factor (BDNF) via Nur77 and ARC through a novel pathway in rat cortical neurons [166]. The glucocorticoid receptor (GR) and MEF-2A are the two key transcription factors involved in neuronal plasticity, and they both modulate c-JUN, a transcriptional target regulating the synapse strength and number. MEF-2A is hyper-phosphorylated by GR activation and in turn regulates c-JUN, which increases MEF-2A DNA binding ability. This molecular cross-talk controls genes of neuronal plasticity [167]. SENP2 (Sumo specific protease-2) post-translationally regulates MEF-2A in post-synaptic differentiation, which is de-SUMOylated and acetylated at Lys403. This modification inhibits dendritic claw differentiation via activation of MEF-2A. SENP2 is highly expressed in the cortex and hippocampus and regulates sumoylation status of MEF-2A for transcriptional activation [129]. Interestingly, sumoylated-MEF-2A is a transcriptional repressor which drives the suppression of orphan presynaptic sites. Synaptogamin (Syt1) was identified as the direct repressed target of sumoylated MEF-2A in neurons. Syt1 eliminates the orphan presynaptic sites and aids in the accumulation of presynaptic material in the maturing boutons, which may be implicated in the neuronal connectivity, brain development, and disease [168]. Formal thought disorder is an important feature of schizophrenia and other psychotic disorders. In a cohort study of high-risk family subjects, microsatellite linkage analysis and whole genome sequencing identified causative variants in the linkage region and reported a MEF-2A binding site that was located between two genes associated with schizophrenia; the locus 6q25-26 has been implicated in formal thought disorder via dysregulated MEF-2A signaling [169]. Also, it has been implicated in oxidative stress during aging and other neurodegenerative disorders that result in neuronal death. Chaperone mediated autophagy (CMA) targets MEF-2A to the lysosomes for CMA degradation, and stress-induced destabilization of the lysosome results in the disruption of MEF-2A and its function thereby leading to neuronal damage in various neurodegenerative diseases [170]. MEF-2A was detected in patient subjects with temporal lobe epilepsy (TLE) and it was significantly downregulated in the temporal neocortex of both humans and rats with TLE compared to control groups, suggesting a role in the pathogenesis of TLE [171].

### MEF-2A mutations and activity control

Contrasting mutational and functional analysis of MEF-2A have been reported in many studies which identify MEF-2A as a susceptibility gene for premature myocardial infarction and coronary artery disease (CAD). Specifically, a 21-bp deletion in exon 12 and 3 and other missense mutations which affect its transcriptional activity have been implicated, but most studies have not demonstrated significance for supporting this hypothesis [172]. Cohort studies performed for Exon 11 deletion examined in CAD subjects reveal that only 0.09% of the population in the Sicilian cohort was reported to have CAD-associated genes compared to healthy subjects [173]. Similarly, in a Chinese-HAN cohort, they found 4 single nucleotide polymorphisms in Exons 9 and 11, one insertion mutation in Exon 11, one deletion mutation in Exon 11, and one STR in Exon 11; however, these structural changes in Exon 11 of MEF-2A did not relate to the sporadic CAD [174]. Similar cohort studies in Chinese Han subjects claim that the 3′ UTR may consist of functionally relevant nucleotide changes, and 2 single nucleotide polymorphisms were detected but deemed not significant, but a further haplotype carrier of rs325380-rs325381 was claimed to be associated with CAD risk, suggesting variance in the 3′ UTR of MEF-2A [175]. Another study in the Saudi population revealed MEF-2A as the susceptible gene for the risk of CAD, upon analysis of Exon 11 which revealed several substitution polymorphisms, insertions/deletions at the 11 CAG trinucleotide loci, which introduced premature stop codons at 4 nucleotide sequences nt146637, nt146647 and nt146780 or nt146783 [176].

Exogenous control of MEF-2A can be regulated by exercise. Many genes are upregulated during exercise and confer protection against diseases such as type 2 diabetes. Studies have shown that NRF-1, known to regulate mitochondrial oxidative genes, can also affect MEF-2A and GLUT-4. Exercise-induced CAMKII activation induced hyperacetylation of histones of both *NRF-1* and *MEF-2A* genes and increased glucose transport by upregulation of GLUT4 in rats [177]. Similar exercise regimens performed in mice, where muscle-type carnitine palmitoyl l1 (CPT1b) is involved in skeletal muscle mitochondrial β-oxidation, suggest binding of MEF-2A to the Cpt1b promoter, which elevates the growth of the quadricep muscles and development of skeletal muscle; binding activity was decreased with exercise training and increased expression of HDAC5 which correlates to the decrease in *MEF-2A* activity [178]. Additionally, other studies indicate that exercise increased nuclear *MEF-2A* content and increased binding of *MEF-2A* to the GLUT-4 gene in a AMP activated protein kinase (AMPKα2)-dependent mechanism [179].

### Infection, immunity and cancer

MEF-2A has been implicated in various disease models ranging from neuronal to tumorigenic. It has contrasting roles in various tumor types and has been linked to various disorders such as hepatocellular carcinoma (HCC), gastric cancer, atherosclerosis and also in immunity. TGF-β1 (transforming growth factor β1) is involved in HCC invasion, and *MEF-2A* along with other isoforms are overexpressed in HCC cells in a PI3K/AKT-dependent manner. By modulating TGF- β1 signaling, MEF-2A promotes epithelial to mesenchymal transition (EMT) [180]. TGF-β is also a transcriptional activator of Matrix metalloprotease-10 (MMP-10) via activation of MEF-2A and downregulation of Class IIa HDACs [181]. In gastric cancer MEF-2A is phosphorylated by P38 to activate glycolysis via the GLUT-4 transporter [182]; moreover, MEF-2A mRNA is overexpressed in 10% of tumors of patients with gastric carcinomas. Recently, MEF-2A has been identified as a proapoptotic factor in therapeutics of HCC via activation of caspase-3 and caspase-7, and thereby inhibiting growth of HCC xenografts in nude mice [183]. MEF-2A has been implicated in the progression of pre-existing atherosclerotic lesions. The inflammation patterns of apolipoprotein E-deficient mice following the knockdown of MEF-2A showed accelerated atherosclerosis [184]. Our lab was the first to implicate MEF-2A as a regulator of viral gene expression in the pathogenesis of HTLV-1. HTLV-1 encodes the oncogenic trans-activating Tax protein, which directly binds to MEF-2A and hijacks the host transcriptional machinery to increase viral gene expression [17]. MEF-2A expression was upregulated in the peripheral blood of HTLV-1-infected individuals. We are currently studying other isoforms of MEF-2 and their implications in HTLV-1-induced ATLL (unpublished observations). MEF-2A has been reported in the differentiation of many cell types. MEF-2A and MEF-2D play dual roles in human macrophage terminal differentiation by forming heterodimers with each other and activating P300 binding and c-JUN expression in differentiated macrophages [8].

### MEF-2B

MEF-2B is the most distant member of the MEF-2 family of transcription factors based on phylogenetic analysis, and it also lacks the HJURP_C (Holliday junction recognition protein C-terminal) region and other duplication events [185]. In the apo structure of MEF-2B (only MADS-box/MEF-2 domain) without DNA or cofactor binding, it showed a preformed DNA binding interface that may be important for recognizing the DNA from the minor groove side and the C-terminal helix which can serve as the docking site for MEF-2 transcription co-factors suggesting new interaction patterns [186]. This gene undergoes considerable alternative splicing events. MEF-2B is the only isoform that is not ubiquitously expressed, but is enriched only in bone marrow and immune cells and the gene is located on chromosome 19p12. At the transcriptome level it is expressed in the appendix, bone marrow, and tonsils at 17.6 tpm, 45.7 tpm and 27.2 tpm, respectively. High protein expression of MEF-2B is only found in the germinal centers of lymph nodes and tonsils, but not in non-germinal centers or in other cell types. Low or no expression of MEF-2B is observed in many cancer types and is significantly downregulated in lymphomas and lung carcinomas (https://www.proteinatlas.org/).

### MEF-2B in development

MEF-2B is also involved in developmental programs and its mutations have been implicated in the genesis of leukemias and carcinomas. In smooth muscle specific gene expression, the *SMHC* (smooth muscle myosin heavy chain) gene consists of an A/T rich region which provides a binding site for MEF-2B, and overexpression of MEF-2B increased SMHC activity suggesting its role in SMHC gene regulation [187]. MEF-2B modulates inducible expression of NOX-1 in superoxide production in various cardiovascular tissues via ATF-1:MEF-2B signaling [45]. During cyclic stretch (CS) in VSMCs, ROS (reactive oxygen species) are produced via MEF-2B:NOX1 signaling causing dysfunction of VSMCs by switching from contractile to synthetic phenotype leading to hypertension [188]. During mouse embryogenesis, MEF-2B transcripts were expressed in cardiac and skeletal muscle and its gene mapped to chromosome 8, and its potent transactivation was controlled in the C-terminal domain [189].

MEF-2B is widely implicated in cancer phenotypes and poor prognosis based on its mutations and the pathways it regulates. In the P19 teratocarcinoma murine stem cell line, MEF-2B homologue is differentially expressed during development and is predominantly found in non-committed cells [190]. In a kinome-wide siRNA screen, the MEK5-ERK5 signaling axis was implicated in the activation of MEF-2B, thereby leading to EMT, lung metastasis and invasion in breast cancer cells. This MEK5-ERK5:MEF-2 axis promoted TGFβ-induced EMT in murine and human breast cancer cells. MEF-2B drastically affects the morphology of the cell and gene expression patterns during EMT [191]. Similarly, EGF activates the ERK5:MEF-2B pathway which induces the expression of DDAIS, an anti-apoptotic protein activated during DNA damage, and promotes tumor invasion by activating the β-catenin signaling pathway. MEF-2B binds to sequence specific promoter binding sites in DDAIS which activates the ERK5/MEF-2B pathway for promoting tumorigenesis [125].

### Extensive MEF-2B mutations in lymphomas

Mutations in MEF-2B have been shown to have fatal consequences in different disease phenotypes. MEF-2B is a target for somatic mutations in various lymphomas, most of these affecting the MADS-box/MEF-2 domain, and the most frequent being the D83V mutation in cancer. Crystallographic analysis revealed that most mutations are non-synonymous substitutions altering the structure and function of the protein, causing changes in the MEF-2 domain from an α-helix to β strand with resulting binding defects in various transcriptional co-factors. This conformational switch is a key mechanism in NHL (Non-Hodgkin's lymphoma) in driving tumorigenesis [192]. MEF-2B acts as a transcriptional activator and is mutated in 11% of Diffuse large B-cell lymphomas (DLBCL), and 12% of follicular lymphomas. BCL6, which is a direct target of MEF-2B, controls germinal center B cells. The oncogenic mutations in MEF-2B largely impact the dysregulation of the BCL6 proto-oncogene which controls the cell cycle and B-cell differentiation. Mutation in the N-terminal domain results in a gain of function phenotype by preventing the binding of CABIN-1, a corepressor of MEF-2B. C-terminal truncations, nonsense, or frameshift mutations might affect the PTMs and transcriptional activity of MEF-2B [193]. BCL6 is also regulated by AhR (Aryl hydrocarbon receptor)/ARNT complex and wild type MEF-2B and if mutated may cause DLBCL [194]. Also, similar studies indicate hotspot mutations in K4E, Y69H and D83V in DLBCL affecting DNA binding and functional activation respectively [195]. Comparative analysis of MEF-2B with other germinal center antigens revealed that in differential diagnosis of B cell non-Hodgkin's lymphoma there was positive expression of MEF-2B in all FL (follicular lymphomas) and BL (Burkitt's Lymphoma), 8/9 of mantle cell lymphomas and 2/24 of Splenic marginal zone lymphomas (MZL) and 38/44 DLBCLs but negative expression in extranodal MZL and B-lymphoblastic lymphomas [196]. Genetic alterations were reported in FL and revealed 85% translocations [197], 96% mutations in the *BCL2* gene and about 15% frequency of genetic alterations in MEF-2B. In an extensive analysis of various B and T-cell lymphomas to validate MEF-2B as an immunohistochemical marker, the expression revealed a statistically significant association with BCL6 in DLBCL and indicates MEF-2B as a valuable marker for differential diagnosis of various types of lymphomas [193]. Clinical interventions via HDACi that function as major repressors of MEF-2B activity have been reported. Pabinostat which is a Pan-HDACi was used to treat relapsing DBLCL subjects in a phase II trial along with/without rituximab showed durable responses in 28% of the patients and early responses were predicted for MEF-2B and significant increase in the ct-DNA was a surrogate for subsequent treatment failure [198].

### MEF-2C

MEF-2C is one of the most characterized genes in the MEF-2 family and has shown to be involved in various neural, cardiac, skeletal, muscle, lymphoid and myeloid developmental programs, and has also been implicated in various types of T-cell and B-cell lymphomas, carcinomas, many neurodegenerative diseases and vascular disorders. MEF-2C has transactivation and DNA binding domains and its gene is located on chromosome 5q14.3. It is highly expressed in the nucleoplasm and nucleoplasmic vesicles. Although it is ubiquitous in nature, its enrichment is limited to specific cell types found in the brain and muscle tissues. The highest expression of MEF-2C mRNA transcripts is found in the cerebral cortex of the brain (197.9 tpm), but is absent in the hippocampal and cerebellar regions. Also, similar expression is observed in skeletal muscle (192.4 tpm). MEF-2C is perhaps the only member of the family where the transcriptome expression correlates with the protein expression patterns, both in the CNS and skeletal muscle, with high levels of protein expression observed in these tissue types. Furthermore, MEF-2C protein expression is similarly abundant in the immune system where higher expression is observed in the lymph nodes, tonsils, and spleen, but surprisingly not in the bone marrow but considerably less at the transcript level at 75.6 tpm, 70.6tpm, and 60 tpm, respectively. MEF-2C is highly overexpressed in lymphomas, melanomas and gliomas (https://www.proteinatlas.org/).

### MEF-2C in development

MEF-2C is the predominant isoform regulating the development of the cerebrocortex, and conditional knockout of *MEF-2c* in mice in neural stem cells (NSCs) abolished neuronal differentiation. These findings suggest critical roles of MEF-2C in early neuronal differentiation and proper distribution of layers in the neocortex [146]. Activation of MEF-2C-dependent Bone Morphogenetic protein2 (BMP2) by P38 MAPK controls the commitment switch between cardiomyocyte and neuronal commitment. Inhibition of P38 MAPK in ES cells (Embryonic stem cells) results in the neuronal commitment of the ES cells; similarly, treatment with BMP-2 in ES cells results in the cardiomyocyte differentiation which is regulated by MEF-2C, suggesting a molecular mechanism for ES cell commitment [199].

Proper neurite function and outgrowth are necessary for neuronal function and synapse formation. miRNAs play an important role in neuronal development and function, and miR-124 and miR-9 are abundantly expressed in the mammalian nervous system. HDAC5 inhibits neurite extension by inhibiting the neuronal membrane glycoprotein GPM6A(M6a), which is regulated by MEF-2C. miR-124 and miR-9 modulate this HDAC5:MEF-2C:M6a pathway to regulate neurite development in primary neurons [200]. BDNF is a neurotrophin which controls synaptic development and function, and it regulates many signaling pathways leading to activation of MEF-2 family members. MEF-2C splice variants which lack the *γ-domain* are activated by membrane depolarization, and the knock down of MEF-2C resulted in the impairment of membrane depolarization-induced expression of *Bdnf* exon 1, suggesting the differential activity of MEF-2C [201]. Cortico-basal ganglia circuits control language and speech in Autism spectrum disorders (ASD). FOXP2 is known to interact with several ASD related genes and have been implicated in spoken language disabilities and dysfunction in the corticostriatal circuit. MEF-2C, which is a synapse suppressor negatively interacts with *Foxp2.* The deletion of FoxP2 derepresses MEF-2C function, which then restores vocalization and related striatal spinogenesis suggesting MEF-2C function in corticostriatal circuit [202]. MEF-2C is abundantly expressed in cortical excitatory neurons, and conditional knockdown of *MEF-2c* in hippocampal and cortical excitatory neurons decreases the cortical network activity by decreasing the excitatory synaptic transmission. *MEF-2c* mutants showed significant overlap of synaptic and autism-linked genes in the cortex and mutant mouse models displayed autism and schizophrenia-like behavior, suggesting functional importance of MEF-2C in the neo-cortex [10]. Constitutively expressing MEF-2C in both *in vivo* and *in vitro* systems yielded pure neurons and MEF-2C-directed neuronal progenitor cells can successfully differentiate into functional neurons in mouse models of cerebral ischemia and can improve behavioral defects [146]. The haploinsufficiency of MEF-2C reported in patient and mouse models leads to severe mental retardation, epilepsy and cerebral malformations, and also the mice exhibited severe hyperkinesis. Moreover, MEF-2C was implicated in various developmental stages of dorso-ventral neuronal cell types [203]. A microdeletion involving the 5q14.3 region of the chromosome resulted in the haploinsufficient phenotype of MEF-2C [204]. Similar clinical studies reported that decreased MEF-2C mRNA transcripts in leukocytes may serve as a diagnostic marker in Alzheimer's disease (AD), and this may also be associated with the decline in cognitive ability [205]. Rett's syndrome is characterized by severe mental retardation, epilepsy, absence of speech and cerebral malformations, and MEF-2C polymorphisms have been found in patients with Rett's or severe Rett-like encephalopathies [206].

### MEF-2C in diseases

Various congenital diseases arise from the improper development of the neural crest cells (NC cells) which undergo various developmental changes during morphogenesis, including craniofacial defects. MEF-2c NC knockout mice exhibit delayed ossification and hypoplasia (loss of skeletal elements of the face and skull). Dlx6 and Hand2 genes are responsible for these developmental pathways, where MEF-2C is the direct transcriptional regulator of Dlx6-Hand2 and affects craniofacial development via a feed-forward transcriptional circuit mechanism in mice [207]. In addition, Endothelin, which is also essential for neural crest development, targets MEF-2C downstream of a calmodulin-CamKii histone deacetylase signaling cascade through a positive feedback mechanism [208].

TBX-1 is a T-Box transcription factor that has been implicated as a haploinsufficient gene in DiGeorge syndrome, which is characterized by congenital defects of the heart, craniofacial dysmorphism, abnormal thymus gland and hypoplasia. *Tbx* null mutant embryos showed higher MEF-2c expression, and conversely MEF-2c expression was decreased in the Tbx1 gain-of-function mutants, revealing the suppressive effects of Tbx on MEF-2c. It was also shown that Tbx1 interferes with the Gata4 signaling regulation of MEF-2c affecting critical developmental pathways [209]. Conversely, TBX5 plays a synergistic role with MEF-2C in the early phases of cardiomyocyte development and differentiation, and mutations in TBX5 are associated with Holt-Oram syndrome which features massive heart and forelimb abnormalities [210]. During embryogenesis, the binding of MEF-2C in the BOP gene promoter is responsible for *bop* expression in the anterior heart field and its cardiac derivatives [211]. Similarly, transcription factors *NKX 2.5* and *MEF-2C* bind physically and functionally to control ventricle formation during cardiogenesis [212], and also are required for the maintenance of the cells between the ventricular and sinoatrial precursors in the primary heart field [211]. However, in certain mouse models, knockdown of *MEF-2c* attenuated left ventricular hypertrophy and mechanical stress via the mTOR/S6K pathway [213]. The specific isoform MEF-2cα1 is involved in dynamic phosphorylation events implicated in myoblast and skeletal muscle regeneration via Akt/mTOR/S6K signaling which may potentially be targeted for treatment of muscle wasting diseases [214]. There is evidence for miRNA regulation of MEF-2C; miR-214 suppresses the activity of MEF-2C:MYOCD:Leiomodin1(LMOD-1) signaling pathway, and excessive proliferation of smooth muscle cells causing pulmonary arterial hypertension [215]. Dysregulated expression of several miRNAs and mRNAs in a myotonic dystrophy type 1 (DM1) cell model, was rescued with the exogenous addition of MEF-2C [216]. Hormonal mediators such as testosterone and IGF (Insulin like growth factor) also modulate MEF-2C in cardiomyogenesis and pro-hypertrophic effects in cardiac gene function via HCN4 and P38-MAPK respectively [217]. MEF-2C interacts with the transcription factor c-FOS and increases its binding to an AP-1 site in the Mmp13 promoter, which is modulated by para-thyroid hormone (PTH), a transcriptional regulator of many genes in osteoblast formation [218]. Another known co-repressor of MEF-2c is HDAC-5, whose increased expression decreases osteocyte formation. Binding of MEF-2C in the sclerostin, encoded in the SOST gene, after its detachment from the repressor complex, derepresses MEF-2C function in the direct regulation of osteocyte formation via SOST gene expression [219]. Also, studies reveal that MEF-2C/NFAT pathways along with GATA4 that juncture the Ca2+ signaling have been implicated in cardiac hypertrophy and heart failure from a cohort of heart transplant patients [220]. Similarly, in a study of 200 patients with congenital heart defects (CHD), loss of function mutations in MEF-2C were reported to be associated with CHD, which also affects the activation of transcription factors such as GATA4 [221]. MEF-2C also controls circadian clock rhythms in Drosophila models [222]. Aquaporin is present in the microvascular endothelial cells and is responsible for formation of water channels that control various physiological processes; *MEF-2C* is a direct target of Aquaporin which controls angiogenesis and water processes in endothelial cells [223].

### MEF-2C in cancer and immunity

MEF-2C is associated with the development of various cell types including myeloid and lymphoid progenitor cells, and its deficiency results in profound defects in the phenotypes of both cell types. It has been also implicated in fate commitment of the progenitor cells, determining if a cell will differentiate along the myeloid or lymphoid lineage [149]. Deficiency of *MEF-2c* results in delayed B-cell development with significant reduction of immature B cells; *MEF-2c* deficiency also alters the expression of various proteins including CD23, OX40L and Ciita [224]. Moreover, MEF-2C is required for the survival and proliferation of B-cells and B-cell signaling receptor function [148], and also modulates B-cell proliferation via P38 MAPK activity and EBF1 (Early B cell factor 1) [107]. MEF-2C regulates megakaryopoiesis [225], and via a MEF-2C/Nur77 pathway mediates activation-induced cell death of macrophages [226]. The activation of MEF-2C by MEKK-2 kinase activity induces c-JUN expression and activates cytokine production through FcἐR1 for stimulation of mast cells [227]. MEF-2C is also required for restraining age-related microglial inflammation through excessive production of IFN-beta and its loss results in brain related inflammatory syndromes [228]. Although it has been associated with exclusively B-cell restricted transcription factors [229], it also exerts multiple roles in T-cell associated function and disease. MEF-2C is activated by several mechanisms in various T-cell acute lymphoblastic/leukemia cell lines [230]. Transcriptome and molecular-cytogenic analysis, (Chromosome conformation capture on chip (4C)) of >117 pediatric patients revealed the involvement of MEF-2C and NKX2-1 as potential candidates as oncogenes in T-cell acute lymphoblastic leukemia [231]. Additionally, MEF-2C is significantly upregulated in chronic myelogenous leukemia (CML) patients and cell lines. MEF-2C and the CEBP (CCAAT-enhancer binding protein) pathway are associated with disease progression in CML [232].

Loss of skeletal muscle is a characteristic feature and leads to poor prognosis in cancer-associated cachexia; there is a downregulation of MEF-2C (at the mRNA and protein levels) in cachexia which leads to the loss of skeletal muscle architecture and mitochondrial integrity as observed by electron microscopy. This occurs due to the dysregulation of MEF-2C gene targets-myogenin and myokinase, and also downregulated oxygen carrying capacity, ATP regeneration and the calcineurin pathway, culminating in the severe diseased phenotype of contractile muscle and skeletal instability associated with muscle cachexia [233]. Knockdown of MEF-2C in endothelial cells upregulates pro-inflammatory molecules and enhances the adhesion of leukocytes to endothelial cells and activated NF-κB. This process is partially controlled by KLF2 (Krüppel-like Factor 2), and overexpression of MEF-2C has the opposite effect, establishing the function of MEF-2C as a negative regulator of inflammation in endothelial cells [234]. *MEF-2C* is one of the targets downregulated by miR-223, but the expression of *MEF-2C* is negatively correlated with miR-223 in CML patient samples, suggesting its role in CML pathogenesis [235]. Gene expression patterns also reveal that *MEF-2C* is highly overexpressed in pediatric patient samples of ETP-ALL (Early T-cell precursor acute lymphoblastic leukemia) [236]. Alternative splicing of MEF-2C mRNA results in myogenesis and tumorigenic phenotype in Rhabdomyosarcoma (RMS) cells. The inactive myogenic splice variant of MEF-2Cα1 is ubiquitously expressed, but only the active variant MEF-2Cα2 is required for active differentiation. The α exon is aberrantly expressed in RMS cells and overexpression of MEF-2Cα2 resulted in normal myogenesis and differentiation of RMS cells [237].

### MEF-2D

Like all the members of the MEF-2 family, MEF-2D also plays a key role in neuronal cells, muscle cells and other developmental pathways and is regulated by various class II HDACs, specifically HDAC4 and 5. MEF-2D also undergoes alternative splicing which yields multiple transcript variants. The *MEF-2D* gene is located on chromosome 1q12- q23. It is ubiquitously expressed and exhibits high and uniform distribution of protein expression in most-cell and tissue types ranging from brain to skin. Although elevated mRNA transcripts are found in cerebral cortex, bone marrow, and skeletal muscle with 50.9, 49.8 and 55.5 tpms, respectively. All cells and tissues have high levels of protein expression of MEF-2D in cerebral cortex, endocrine system, bone marrow, hematopoietic, germinal centers and also non-germinal centers, cardiac and skeletal muscles, kidneys, breast and skin, etc. MEF-2D is mainly localized in the nucleoplasm and overexpression of MEF-2D is observed in colorectal, breast, prostate, lung and liver cancers, with 100% overexpression in lymphomas, urothelial, breast, thyroid carcinomas and melanomas (https://www.proteinatlas.org/).

### MEF-2D in development

*MEF-2D* null mice experience suppressed cardiac hypertrophy, decrease in fibrosis, and an inability to activate fetal activation [145]. MEF-2D represses skeletal myogenesis upon phosphorylation by protein kinase A (PKA) and HDAC4 activity in the nucleus [57]. Recently, MEF-2D has been implicated in pro-tumorigenic effects, causing cardiac myxoma (CM), the most common cardiac tumor. CM tissue has upregulated expression of MEF-2D, and this is correlated with increased tumor size. MEF-2D regulates IGF-induced proliferation and control of apoptosis in the pathophysiology of CM, and knockdown of MEF-2D reduced the activity of IGF-1 and Matrix metalloprotein 9 (MMP-9) and associated tumorigenesis [238]. Similar studies reveal that MEF-2D expression positively correlates with the proliferation of CM cells; MEF-2D expression can be suppressed by miR-218, a tumor suppressor which is downregulated in myxoma cells and serves as an important target for treatment of cardiac myxoma [239].

Dual-specificity tyrosine-phosphorylation regulated kinase 1A (DYRK1A) is encoded in chromosome 21, which is a critical region associated with Down's syndrome. MEF-2D is involved in the upregulation of this kinase and activates isoform-5 of DYRK1A. The interaction of these genes promotes neurodevelopment [240]. MEF-2D acts as a neuronal survival factor in the maintenance of dopaminergic neurons (DN), the progressive death of which leads to Parkinson's disease (PD). PD-associated neurotoxins destabilize MEF-2D by PTMs. MPP^+^, a toxic metabolite, decreases the half-life of MEF-2D in DA neuronal cell lines by MEF-2D protein degradation and destabilizing MEF-2D mRNA [241]. Another study reports that modulation of transcription factor *Nur77* via *MEF-2D* results in the DN loss in response to another neurotoxin, 1-Methl-4-phenyl-1,2,3,6-tetrahydropyridine (MPTP). The neurotoxin affects the binding of MEF-2D and Nur77 under basal conditions and significantly decreases the expression of MEF-2D and Nur77. This results in the severe dopaminergic loss and increase in the post-synaptic Fos-B activity indicative of nigrostriatal damage in mice [242].

Dysfunctions in chaperone mediated autophagy (CMA) via α-synuclein are also associated with PD. Rotenone, a mitochondrial complex-1 inhibitor, has been used to study CMA substrates. The inhibitor induces the accumulation of α-synuclein and MEF-2D in the cytoplasm due to impaired lysosome-mediated degradation, and affected the protective role of MEF-2D resulting in mitochondrial dysfunction and oxidative stress [243]. The accumulation of α-synuclein in neurons of the nigral pathway reduces the protective activity of MEF-2D in idiopathic and experimental PD [244]. The expression of MEF-2D in microglia modulates neuroinflammation. MEF-2D binds to a MEF-2 binding site in the promoter region of the *IL-10* gene and transcribes IL-10; the silencing of MEF-2D decreased IL-10 induction and increased the levels of TNF-α expression resulting in neuroinflammation [245]. MEF-2D also acts as a potential oncogene in the tumorigenesis of malignant glioma and is highly upregulated in grade 3 and 4 malignant glioma patients [246].

### MEF-2D fusion proteins and disease

RNAseq analysis of 560 ALL patients report the rearrangements of *MEF-2D* along with five other genes. The rearrangements enhance the transcriptional activity of MEF-2D, activated histone acetylase-9, and promoted lymphoid transformation. Additionally, it results in *de novo* fusion proteins with BCL9 and FOXJ2 in a number of cases [247]. Another study with similar RNA-seq analysis of the Philadelphia chromosome reported MEF-2D translocations in the subjects and similar fusions of MEF-2D/BCL9 and MEF-2D/HRNPUL1. MEF-2D/BCL9 fusions are lethal and have the ability to cause leukemia. Many MEF-2D fusion mutations have dire prognosis: most of the mutations cause B-ALL, although some of these fusion products are not completely investigated [41, 147]. Recently, functional studies revealed the pathogenic role of another fusion product resulting in the chromosomal translocation of MEF-2D/SS18 and had the worst prognosis. The studies showed significant inhibition of mouse common lymphoid progenitors into CD19 positive B-cells and diminished B-cell development; additionally, drug sensitivity to B-cell survival is upregulated and plays an important role in the pathobiology of B-ALL [248]. Neurotrophic tyrosine kinases (NTRKs) are highly effective targets for treatment options in many cancers. Fusion proteins involving NTRK1,2, and 3 are being actively investigated in the study of rare cancers such as secretory breast carcinoma, infantile fibrosarcoma, and congenital mesoblastic nephroma. One study reveals the cytoplasmic/membranous expression of NTRK1/MEF-2D fusion protein with a strong (3+) and uniform distribution among carcinomas [249]. Clinical bioinformatic analysis reveals that patients suffering from chronic obstructive pulmonary disease have poor prognosis for developing lung cancer and have higher expression of MEF-2D in non-small cell lung carcinoma (NSCLC), because of its affects on cell proliferation, differentiation and metastasis [250]. In lung cancer, expression of miR-30a has been inversely correlated with MEF-2D, and miR-30a mimetics inhibited the growth of lung cancer by suppressing MEF-2D [251]. The study predicts that miR-30a targets the 3′UTR of MEF-2D mRNA and promotes apoptosis in lung cancer cells. Similar studies performed in osteosarcoma patients reveal that over-expression of MEF-2D and miR-30a leads to a tumor suppressor effect and suppresses osteosarcoma cell proliferation by inhibiting MEF-2D [252]. Long-coding RNAs (lncR-D63785) are emerging trends in cancer implications, and it was revealed that lncR-D63785 expression inversely correlated with miR-422a which can downregulate MEF-2D and drug sensitivity in gastric carcinoma. Knockdown of lncR-D63785 upregulates the expression of miR-422a and sensitized gastric cancer cells to apoptosis induced by doxorubicin; the long non-coding RNA served as a competitive endogenous RNA for miR-422 and promoted chemoresistance by suppressing MEF-2D [253]. MEF-2D is also implicated in angiogenesis and EMT via TGF-β in an autoregulatory mechanism in hepatocellular carcinoma (HCC). MEF-2D along with other isoforms were found to increase expression of TGF-β in HCC, resulting in the activation of the PI3K/AKT/MEF-2 signaling pathway which promoted the progression of metastasis and EMT in HCC [180].

### MEF-2 based therapeutics, HDACi

Class IIa HDACs are involved in the direct binding and suppression of MEF-2 proteins through the MAD/MEF-2 domains [63]. Association of class II HDACs with MEF-2 results in the deacetylation of histones in the vicinity of MEF-2 DNA-binding sites and subsequent suppression of MEF-2 target genes [254]. Furthermore, the association of HDAC with MEF-2 can trigger sumoylation of MEF-2 and further decrease its transcriptional potential [255]. Interestingly, HDAC4 and 5 are highly expressed in the heart, skeletal muscle, and brain, the same tissues in which MEF-2 expression is highest [63]. HDACs 4, 5, 7, and 9 interact with MEF-2 to regulate the differentiation of various cells, including those in bone, brain, skeletal and cardiac muscle, and vascular endothelium [255]. Aberrations in class IIa HDACs are associated with skeletal abnormalities, cardiac defects, denervation super sensitivity, and embryonic lethality [255]. More recently, the HDAC-MEF-2 axis has been implicated in various cancers. Increased expression of class IIa HDACs has been correlated with suppression of MEF-2 transcriptional activity and poor prognosis of estrogen receptor-positive (ER+) breast tumors as well as increased proliferation of mammary epithelial cells [76]. Overexpression of HDAC9 has been linked to poor prognosis of oral squamous cell carcinoma by targeting MEF-2D and repressing expression of MEF-2-dependent genes [77]. Class IIa HDACs in conjunction with MEF-2 have been implicated in leiomyosarcomas as well [41].

Because of their widespread influence on oncogenesis, HDACs have emerged as the target of an entire class of anticancer drugs, known as histone deacetylase inhibitors (HDACi). HDAC inhibitors encompass a diverse group of small molecule drugs that can induce apoptosis, cell cycle arrest, differentiation, and autophagy of cancer cells and promote anti-angiogenic effects [256]. These drugs are predominantly efficacious for leukemias but not solid tumors [257]. Although numerous clinical trials are underway involving HDAC inhibitors, only four have received FDA approval for anticancer drugs: Vorinostat and Romidepsin for cutaneous T-cell lymphomas, Belinostat for relapsed or refractory peripheral T-cell lymphoma, and Panobinostat for multiple myeloma [257]. A fifth molecule known as Valproic acid (VPA) is a weak HDAC inhibitor with a known clinical profile that is commonly involved in clinical trials [257]. HDAC inhibitors complementary to specific amino acid residues on particular HDAC isoforms have been formulated, including HDACs 4, 6, and 7 [257]. Zinc-binding HDAC inhibitors have also been described for class I and II HDACs [257]. HDACi are grouped under 4 different classes on the basis of their chemical structures, hydroxamates, short chain fatty acids, benzamides and cyclic peptides [72].

The precise mechanisms of HDAC inhibitors have not been fully elucidated. Vorinostat (suberoylanilide hydroxamic acid, SAHA) is characterized as a pan-HDAC inhibitor, is a hydroxamic acid derivative (inhibits classes I, II, and III), and is thought to increase activity of the tumor suppressor gene RUNX3 in conjunction with the histone acetylase protein p300. Romidepsin, a depsipeptide, is a selective inhibitor of class I HDACs, specifically HDAC1 and 2 [258]. An *ex vivo* mechanistic study of Romidepsin action in HTLV-1-infected ATL cells found that this drug inhibited transcription factors NF-κB and AP-1 as well as decreased expression of cyclin D2 and Bcl-xL. Belinostat is another pan-HDAC inhibitor and was found to induce apoptosis by TGF-β signaling-dependent down-regulation of survivin [259, 260]. Panobinostat is also a hydroxamic acid derivative and a pan-HDAC inhibitor with ten times the inhibitory activity of vorinostat [261]. It has been shown to reactivate tumor suppressor genes such as p21 and inhibit factors such as Akt and hypoxia-inducible factor 1α [262]. VPA or Valproic acid, a short chain fatty acid derivative has been shown to inhibit class I HDACs with more efficacy than class II HDACs and selectively induce proteasomal degradation of HDAC2 by activating the Ubc E2 ubiquitin conjugase [263, 264].

HDAC inhibitors have been utilized in clinical and pre-clinical trials for treatment of ATL. Vorinostat, Romidepsin, and Panobinostat have shown promising anti-cancer effects in pre-clinical and/or clinical studies in ATL and other T-cell malignancies [99]. Aside from Romidepsin, VPA and pan-HDAC inhibitor AR-42 have been utilized in murine ATL models and shown efficacy [265]. AR-42 was found to decrease Akt, Bcl-xL, and survivin in a prostate cancer model [266]. However, in the context of case studies, due to modest response rates, high rate of cytopenias, and concerns of viral reactivation, it is advised that HDAC inhibitors be used cautiously for treatment of ATL [267]. Nevertheless, a phase 2 clinical trial using panobinostat for relapsed DLBCL found that patients with MEF-2B mutations had a higher chance (likelihood ratio 3.67, 95% CI 1.46-9.19) of a complete or partial response to treatment [198]. Peritoneal fibrosis is a pathological condition that alters peritoneal morphology, causing inflammation. The peritoneum is a continuous monolayer of mesothelial cells. When fibrosis occurs, there is a mesothelial to mesenchymal transition (MMT) which causes invasion. MS-275 is a specific class-I HDAC inhibitor which inhibits cellular invasion and promotes MMT reversal in peritoneal fibrosis [268]. HDACi are also used in various preclinical models of immunological diseases such as EAE (experimental autoimmune encephalomyelitis), asthma, and rheumatoid arthritis [269, 270]. Also, HDACi are used as a “shock and kill” mechanism to reactivate latent HIV and to deplete the viral reservoir [271], and recently Class-I specific HDACi were shown to enhance viral latency reversal and also preserve activity of HDAC isoforms for maximal HIV gene expression to target the virus [272]. The final class of HDACi are the Sirtuins inhibitors, which primarily target Sirt 1 and Sirt 2; nicotinamide is in phase III clinical trial for laryngeal cancer. Sirtinol and Cambinol are drugs in the preclinical stage of investigation, and EX-527 is in phase I and II trial for Huntington's disease and glaucoma [86]. Certain studies demonstrate effects of HDACi in anti-parasitic activity against Plasmodium and Trypanosoma [273]. Recently, HDACi have evolved into chimeric HDACi (chi-HDACi) which simultaneously modulate both HDAC and other targets, using molecular hybridization techniques, drugs that target protein tyrosine kinases, epidermal growth factor (EGF), BCL-ABL tyrosine kinase, or VEGFR along with HDACi were made into chi-HDACi structures which can be used to treat multiple cancer types [274].

Although there are many HDAC inhibitors being studied in preclinical and clinical trials, one of the unique class IIA HDACi, namely MC1568, is highly selective to class IIA HDACs and has been shown to activate the tumor suppressor BRAHMA, that is suppressed by HDAC9 and exhibits cytostatic effect in melanoma cells much greater than Class I inhibitors [275]. MC1568 has been shown to decrease cell proliferation and IL-8 production in human melanoma cells, suppress c-JUN which is a transcriptional target of MEF-2 transcription factors and also suppress the activity of histones 3 and 4, RNA polymerase II and TFIIB to the c-JUN promoter [276]. It is now reported as an anti-influenza drug target that inhibits the activity of HDAC6/8 and decreased expression of viral proteins and mRNAs via the early acetylation of HSP90 [277]. It is a unique molecule that utilizes a distinct mechanism by stabilizing the HDAC4-MEF-2D complex [278] and inhibiting myogenesis [279].

Our lab has been characterizing MC1568 in the context of treatment for HTLV-1-induced ATLL, which is a malignant and intractable T-cell neoplasia. Over 5% of the infected population acquire an aggressive form of non-Hodgkin's lymphoma; and there are no preventive or treatment measures in the form of vaccines or drugs respectively. We have shown earlier the implication of MEF-2A in the viral gene expression and T-cell proliferation and transformation [17]. We are currently investigating MC1568 in the treatment of ATLL, preliminary data suggests that MC1568 is selectively cytotoxic to virus producing leukemic cells but not non-infected cells. Moreover, activated autophagic signaling and a dose-dependent inhibition of viral protein expression of Tax and HBZ was also observed (Madugula et.al unpublished observations). Further mechanistic studies are underway to delineate the involvement of MEF-2 isoforms in the pathobiology of HTLV-1-induced ATLL. MC1568 might be the first of its kind for the treatment of ATLL.

### Conclusions and perspectives

Due to the high-risk disease characteristics associated with MEF-2 aberrations, the outcomes of treatment are less encouraging, the diversity of disease phenotypes caused by MEF-2 family members are very peculiar and are associated to one or more isoforms, but the treatment regimens still more uncertain. Most studies concerning MEF-2 isoforms are based on their gain and loss of function, but the basal level expression of each isoform and how much it is altered in each tissue and cell type is still an open question. Although each isoform has been classified as an oncogene or tumor suppressor, it remains unclear the genetic alterations and molecular aberrations that are occurring in neoplasms associated with MEF-2 isoforms. Due to the differences in the dynamic nature of the MEF-2 isoforms in context of their roles in infection or leukemogenesis, it is warranted to develop isoform-specific molecular targets. Moreover, due to their association with specific classes of HDACs, it would be noteworthy that MEF-2 isoforms would serve as a potential therapeutic target in the treatment of a wide array of diseases.

Epigenetic regulators in the context of therapeutics, especially in the case of cancer and more recently in infection, are a matter of intense discussion. But over time with the evolution of disease categories, HDACi have become more and more indispensable in anti-cancer therapeutics and other infections like influenza and ATLL. Because of their selective cytotoxic effects and induction of autophagy and apoptosis in tumor cells, and feasibility in combinatorial treatments, there are multiple clinical trials based on HDACi as reviewed in [280, 281] that are underway to treat various types of blood cancers and solid tumors and some have reached Phase IV of the trials. The enormous potential of these drug moieties can be tapped out for not only cancer-related syndromes but also in the treatment of infectious diseases. It will be a priority to discover more potent HDACi which are more class and isoform-specific molecules that can provide greater efficacy compared to using more broad-spectrum Pan-HDACi. In this context using Class II specific HDACi that are associated with MEF-2 isoforms may be anticipated to show greater therapeutic potential and fewer off-target effects. Therefore, MEF-2 isoforms can be harnessed by modulating these specific class of HDACs via a specific class of HDACi to open a new range of therapeutics for a wide array of diseases.

## Abbreviations

MEF-2: Myocyte enhancer factor-2
HDACs: Histone deacetylases
HDACi: Histone deacetylase inhibitors
IL-2: Interleukin-2
TCLs: T-cell Leukemias/Lymphomas
T-ALL: T-Cell Acute Lymphoblastic Leukemia/Lymphoma
ATLL: Adult T-Cell Leukemia/Lymphoma
CTCL: Cutaneous T-Cell Lymphoma
PTCL: Peripheral T-cell Lymphoma
EBV: Epstein-Barr virus
HIV: Human immunodeficiency virus
HTLV-1: Human T-cell Leukemia virus-1
NFAT: Nuclear Factor in activated T cells
MITR: MEF-2-interacting transcriptional repressor
IFN: Interferon
VSMCs: Vascular smooth muscle cells
AD: Alzheimer's disease
TPM: Transcripts per kilobase million
Capn-3: Calpain3
mi-RNA: microRNA
BDNF: Brain-derived neurotrophic factor
CMA: Chaperone mediated autophagy
TLE: Temporal lobe epilepsy
CAD: Coronary artery disease
DLBCL: Diffuse large B-cell Lymphomas
CML: Chronic myelogenous Leukemia
KLF: Krüppel-like factor
ETP-ALL: Early T-cell precursor acute Lymphoblastic Leukemia
HCC: Hepatocellular carcinoma
EMT: Epithelial to mesenchymal transformation
TGF-β: Transforming growth factor β
PKA: Protein Kinase A
GSK3 β: Glycogen synthase kinase 3β
